# Life Quality Impairment Caused by Hookworm-Related Cutaneous Larva Migrans in Resource-Poor Communities in Manaus, Brazil

**DOI:** 10.1371/journal.pntd.0001355

**Published:** 2011-11-08

**Authors:** Angela Schuster, Hannah Lesshafft, Sinésio Talhari, Silás Guedes de Oliveira, Ralf Ignatius, Hermann Feldmeier

**Affiliations:** 1 Institute of Microbiology and Hygiene, Charité Universitätsmedizin Berlin, Berlin, Germany; 2 Foundation for Tropical Medicine in Amazonia (FMT-AM), Manaus, Amazonas, Brazil; 3 Institute of Tropical Medicine, Charité Universitätsmedizin Berlin, Berlin, Germany; National Institute of Parasitic Diseases China CDC, China

## Abstract

**Background:**

Hookworm-related cutaneous larva migrans (CLM) is a common but neglected tropical skin disease caused by the migration of animal hookworm larvae in the epidermis. The disease causes intense pruritus and is associated with important morbidity. The extent to which CLM impairs skin disease-associated life quality has never been studied.

**Methods:**

A modified version of the Dermatology Life Quality Index (mDLQI) was used to determine skin disease-associated life quality in 91 adult and child patients with CLM, living in resource-poor communities in Manaus, Brazil. Symptoms and signs were documented and skin disease-associated life quality was semi-quantitatively assessed using mDLQI scores. The assessment was repeated two and four weeks after treatment with ivermectin.

**Results:**

Ninety-one point five percent of the study participants showed a considerable reduction of skin disease-associated life quality at the time of diagnosis. The degree of impairment correlated with the intensity of infection (rho = 0.76, p<0.001), the number of body areas affected (rho = 0.30; p = 0.004), and the presence of lesions on visible areas of the skin (p = 0.002). Intense pruritus, sleep disturbance (due to itching) and the feeling of shame were the most frequent skin disease-associated life quality restrictions (reported by 93.4%, 73.6%, and 64.8% of the patients, respectively). No differences were observed in skin disease-associated life quality restriction between boys and girls or men and women. Two weeks after treatment with ivermectin, skin disease-associated life quality improved significantly. After four weeks, 73.3% of the patients considered their disease-associated life quality to have returned to normal.

**Conclusions:**

CLM significantly impaired the skin disease-associated life quality in child and adult patients living in urban slums in North Brazil. After treatment with ivermectin, life quality normalised rapidly.

## Author Summary

Hookworm-related cutaneous larva migrans (CLM) is a parasitic skin disease common in developing countries with hot climates. In resource-poor settings, CLM is associated with considerable morbidity. The disease is caused by animal hookworm larvae that penetrate the skin and migrate aimlessly in the epidermis as they cannot penetrate the basal membrane. Particularly in the rainy season, the intensity of infection is high with up to 40 larval tracks in an affected individual. Tracks are very itchy and are surrounded by a significant inflammation of the skin. Bacterial superinfection is common and intensifies the inflammation. The psychosocial consequences caused by CLM have never been investigated. We showed that CLM causes skin disease-associated life quality impairment in 91 patients with CLM. Skin disease-associated life quality was significantly impaired. The degree of impairment correlated to the intensity of infection and the number of body areas affected. After treatment with ivermectin, life quality was rapidly restored.

## Introduction

Hookworm-related cutaneous larva migrans (CLM) is a parasitic skin disease caused by the migration of animal hookworm larvae such as *Ancylostoma braziliense*, *A. caninum* or *Uncinaria stenocephala* in the epidermis. The infection occurs when third-stage larvae come into contact with human skin and penetrate into the epidermis. Since animal hookworm larvae cannot penetrate the basal membrane of the human host, they remain confined to the epidermis where they migrate for several weeks or months, and eventually die in situ [1]. CLM is frequent in impoverished rural and urban communities in countries with hot climates [2], [3], [4], [5], [6]. In these settings the prevalence of CLM can reach 4% in the general population and 15% in children <4 years. [6], [7], [8]. CLM belongs to the category of neglected tropical diseases [9], [10].

The main symptom of CLM is severe pruritus, which intensifies at night. The itching leads to sleep disturbance and day somnolence [6]. Scratching may cause extensive excoriations and subsequent bacterial superinfection of the lesions, typically by *Streptococcus pyogenes* or *Staphylococcus aureus*. Bacterial superinfection by group-A streptococci may induce the development of post-streptococcal glomerulonephritis [11].

A recent study on knowledge, attitudes and practice among mothers of children with CLM highlighted the psychosocial stress associated with this parasitic skin disease and its negative impact on family life (H.Lesshafft 2010, unpublished data). This prompted us to investigate the impairment of skin disease-associated life quality in patients with CLM in a semi-quantitative manner.

## Materials and Methods

### Study Area and Population

The study was carried out in Manaus, the capital of Amazonas State, North Brazil, from October 2008 to February 2009. Patients were actively recruited in resource-poor neighbourhoods, so called *invasões*. Patients were identified via word-of-mouth advertising through primary health care centres, neighbourhood organisations and community leaders. Twenty-three patients were recruited in *Barrio da União* and 28 in *Nova Vitória*; 40 patients came from five further resource-poor communities scattered in the city of Manaus. All communities were situated along small tributaries of the Amazon River (*igarapés*).

In these communities, most houses are built on stilts (*palafitas*) and made of wooden planks or recycled materials. Streets are unpaved, access to drinking water is precarious, sanitation is deficient and garbage is usually disposed in the adjacent *igarapé* or on the street. Dogs and cats stray around and feed on garbage found below and around the houses. In the rainy season, the communities are regularly inundated and animal faeces are widely dispersed.

Usually, households include two to six children. Blended family constellations, single mothers, adult illiteracy and unemployment are frequent. Alcoholism, psychological and physical violence and drug abuse are common.

The setting in which the study was carried out shares many social and economical characteristics with numerous other impoverished urban communities in South America. Most households in which the patients lived benefitted from the national *Bolsa Familia* and *Bolsa Escola* programs which support families with a monthly per capita income <140 Brazilian Reais (equivalent to 54 Euros at the time of study) with regular financial contributions.

### Study Design

The study is part of a larger research project on the epidemiology, morbidity, and control of CLM in North Brazil. Individuals aged ≥5 years with a diagnosis of CLM were eligible for the study. The investigation was performed as a prospective study with active case detection. Pregnant women and children <5 were excluded from the study because ivermectin treatment is contra-indicated in these groups. The study took place between October 2008 and July 2009.The diagnosis of CLM was made clinically. The whole skin was examined in a room where privacy was guaranteed and good lighting was available. The genital area was only inspected when the patient or his/her carer gave verbal consent. Children were always examined in the presence of their mothers. CLM was diagnosed when the characteristic elevated linear or serpingious track was visible and the lesion had moved forward during the preceding days [6], [12]. The number and the topographic localisation of each lesion was documented. Each track was defined as a single lesion, irrespective of the distance between the tracks. Tungiasis (jigger flea) and scabies, parasitic skin diseases also characterized by itching skin lesions, were excluded by careful clinical examination.

In order to determine the topographic distribution of the lesions and the affected area of the skin, the body surface was divided into right and left. As in previous studies each side was subdivided into 14 areas as follows: head, upper arm, forearm, hand, thorax, abdomen, back, buttock, genital/inguinal area, thigh, lower leg, ankle, back and sole of the foot [13]. Body areas were further classified into clearly visible areas (head, forearm, hand, lower leg, back and sole of the foot), partially visible areas (upper arm, thorax, abdomen, back, thigh) and non-visible areas (buttock, genital/inguinal area) according to local dress codes. Lesions were differentiated into papular, crusted-papular, and nodular [13]. The presence and dimensions of excoriations were documented. A simple lesion was defined as a track without bacterial superinfection, excoriations, or an significant inflammation presenting nodular lesion or an extended erythema. Bacterial bacterial superinfection was diagnosed when pustules, suppuration, or an abscess were present [6].

The severity of CLM was determined semi-quantitatively, using a severity score. This score combines the following variables: number of tracks (1–2 tracks = 1 point, 3–5 tracks = 2 points, 6–10 tracks = 3 points, >10 tracks = 4 points); presence/absence of secondary infection (0/2 points); signs of local inflammation (erythema, warmness or swelling = 1 point, pain = 2 points, nodular lesions = 3 points); presence of lymphadenopathy proximal to the lesion (0/1 point). Hence, the severity score can vary between 1 and 10 points.

Immediately after diagnosis patients were treated with ivermectin (200 µg/kg) in a single oral dose (Revectina; Solvay Farma Ltda, São Paulo, Brazil). Two and four weeks after treatment, the patients were re-examined and the mDLQI was determined again.

### Dermatology Life Quality Index

The Dermatology Life Quality Index (DLQI) was developed by Finlay and Khan in 1994 [14]. It is a validated instrument to assess skin-associated life quality impairment and it is the most frequently used tool to determine skin disease-associated life quality in patients with skin diseases of infectious and non-infectious origin [15], [16], [17].

The original DLQI questionnaire is available in English and in several other languages (www.dermatology.org.uk). In the present study, the Brazilian Portuguese translation was used. First, the wording was adapted to local culture and attitudes according to guidelines described by Cestari et al. [18]. Second, the questions were modified to focus on characteristic sequelae of parasitic skin diseases, and their impact on life quality in the setting of resource-poor communities in Brazil. Third, questions not applicable to children, such as the impact of skin disease on sexual life, were omitted in accordance with the original questionnaire for children [19]. This resulted in a modified dermatology life quality index (mDLQI) with eight items and a score varying between 0 and 24 points. The items were the following: pruritus, sleep disturbance, feeling of shame, need to adapt clothing in order to cover up skin lesions, problems faced at work or in school, impairment of leisure activities, impairment in personal relationships, teasing (only children), and problems concerning sexual relationships (only adults). The mDLQI has been validated by Worth et al. in patients with scabies living in a similar setting in northeast Brazil [20].

Since illiteracy was widespread, each statement was read out loud to the patient by one of the investigators (AS or HL) and its meaning explained in a standardized manner. The answers to each statement were weighted as follows: not at all = 0 points, a little = 1 point, quite a lot = 2 points, very much = 3 points [14]. The points for each statement were added up and formed the mDQLI for each patient. The mDLQI scores were categorised as shown in table 1.

**Table 1:** Categories of the modified Dermatology Life Quality Index.

DLQI points	Effect on patient's life
0–1	None
2–3	Small
4–8	Moderate
9–16	Large
17–24	Very large

### Statistical Analysis

The data were entered twice into a database using Epi Info software package Version 3.4.3 (CDC Atlanta, USA) and checked for errors which may have occurred during data entry. Data analysis was performed using SPSS for Windows (Version 16.0; SPSS Inc., Chicago, Illinois). Since data did not follow a normal distribution, the median and the interquartile range (IQR) were used as an indicator of central tendency and dispersion of the data, respectively. The Spearman rank correlation coefficient was calculated for correlations between mDLQI scores and other ordinal variables. The Mann-Whitney-U test was used to compare mDLQI scores between subgroups of patients. Relative frequencies were compared using the chi-squared test.

### Ethical Considerations

The study was approved by the Ethical Committee of the Fundação de Medicina Tropical do Amazonas (FMT-AM), the reference institution for tropical diseases of Amazonas State.

The objectives of the study were explained to each participant in simple and comprehensible Portuguese. The right to withdraw at any time was described in plain words. Patients had time to meditate about their decision and were given the possibility to discuss any doubts with the researchers. Each participant, or in the case of minors, their legal guardian, signed the written informed consent form. In case of illiteracy consent was given via fingerprint. The informed consent form was written and read out loud, and after each paragraph, the participant was asked whether she/he understood its meaning. Patients with other skin diseases than CLM were referred to the nearest primary health care centre or to the outpatient department of the FMT-AM, where treatment was provided free of charge.

## Results

**Figure 1:**
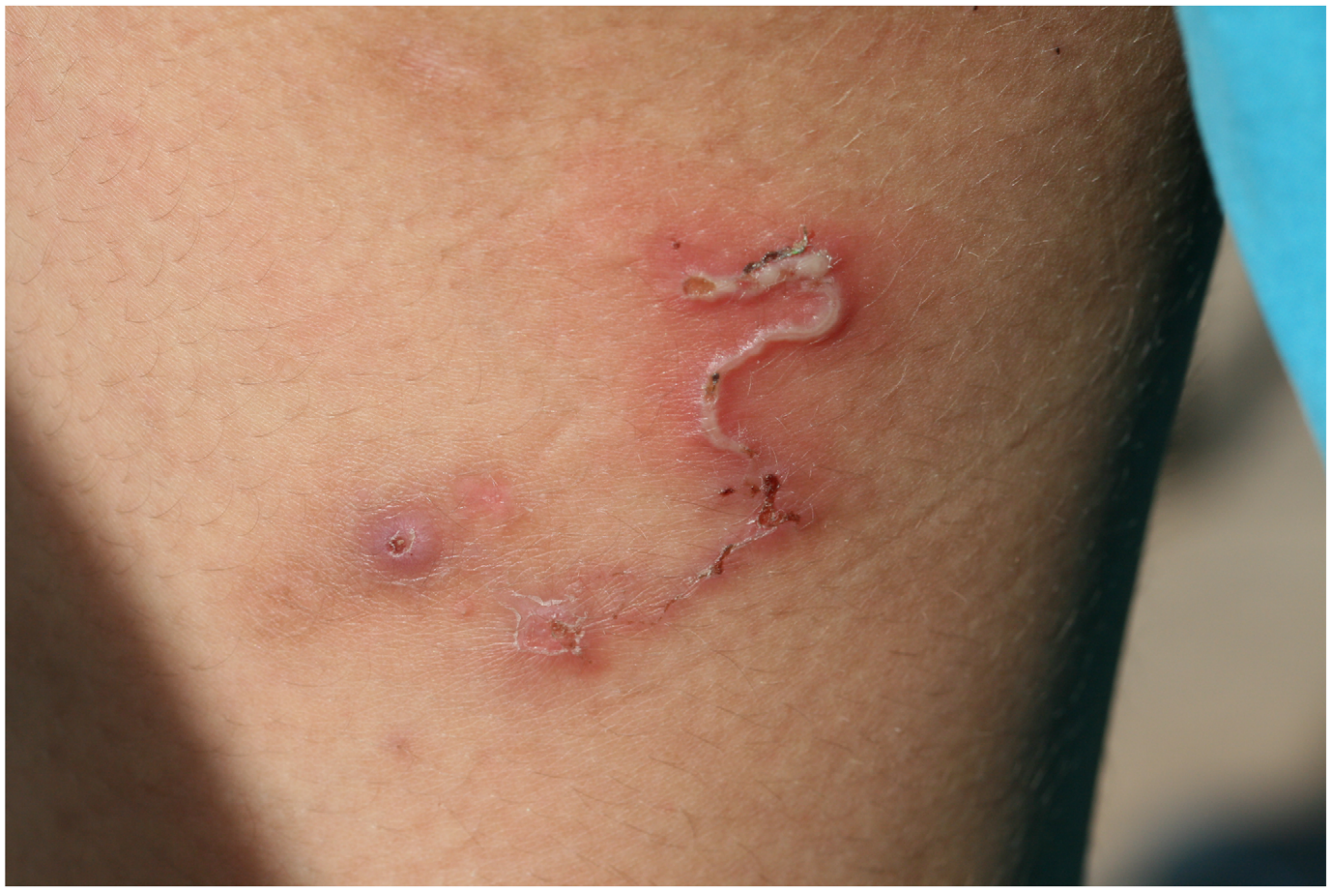
Grossly inflamed and superinfected larval track on the thigh of a 8-year-old boy.

Ninety-one patients were included in the study, 63 of them were male and 28 female. The median age was 10 years (IQR 7-12, range 5–44 years). The demographic and clinical characteristics of the patients are summarized in Table 2. Forty-four point eight percent of the patients had more than two lesions. The maximal number of lesions was 51. 88% of the patients had noted the appearance of the oldest track during the last four weeks. Figure 1 shows a typical example of an inflamed and superinfected track at a visible body part.

**Table 2:** Clinical and demographic characteristics of patients with CLM (n = 91).

Characteristic	N (%)
Sex	
Male	63 (69.2 )
Female	28 (30.8 )
Age (years)	
Median	10
IQR	5–44
**Type of lesions present** a	
Simple track	87 (94.6)
Crusted papular	57 (62.0)
Papular	6 (6.5 )
Nodular	7 (7.6 )
Excoriation	45 (48.9)
Bacterial superinfection^b^	12 (13.0)
**Number of lesions**	
1–2 lesions	48 (52.2)
3–4 lesions	16 (17.4)
5–6 lesions	6 (6.5)
7–8 lesions	8 (8.7)
>10 lesions	14 (15.2)
**Topographic area of the lesions** a	
Uncovered body areas	95 (84.1)
Partly covered body areas	34 (30.1)
Covered body areas	21 (18.6)
**Duration of infection** c	
1–7 days	34 (37.0)
8–28 days	47 (51.0)
>28 days	11 (12.0)

a multiple classifications possible.

b pustules, suppuration, abscess.

c in case of multiple lesions, appearance of the oldest.

Nearly all study participants showed a reduction of life quality (mDLQI≥2 points) at the time of diagnosis (Table 3). The majority of the patients (51.6%) showed a moderate life quality impairment.

**Table 3:** Dermatology life quality impairment in patients with CLM (n = 91).

mDLQI categories	N	%
No effect (0–1 points )	5	5.5
Small effect ( 2–3 points)	21	23.1
Moderate effect (4–8 points)	47	51.6
Large effect (9–16 points )	18	19.8
Very large effect ( 17–24 points)	0	0

At baseline, the median mDLQI score was 5 (IQR 3-8). 6 (IQR 3-9) for adults and 5 (IQR 3-8) for children (p = 0.7; Table 4). Pruritus, sleep disturbance, feeling of shame and the need to dress differently were the most frequent restrictions. Significant differences in perceived restrictions between adult and child patients existed for problems faced at work/school and impairment in social relationships (p = 0.040 and p = 0.026, respectively). There was no difference in mDLQI scores between boys and girls (5 [IQR 3-8] versus 6 [IQR 3-7]; p = 0.86) and men and women (6 [IQR 3.-9] versus 4 [IQR 2-9]; p = 0.63).

**Table 4:** Impairment of life quality in adult and child patients with CLM (n = 91).

Area of impairment	All patients ( n = 91)	Adults (n = 11)	Children (n = 80)	Children vs. adults p-value
Pruritus	85 (93.4)	11 (100.0)	74 (92.5)	0.357
Sleep disturbance	67 (73.6)	9 ( 81.8)	58 (72.5)	0.511
Feeling of shame	59 (64.8)	6 (54.5)	53 (66.3)	0.446
Need to dress differently	31 (34.0)	3 (27.3)	28 (35.0)	0.612
Problems faced at work/in school^a^	13 (14.9)^b^	3 (42.9)^c^	10 (12.5)	**0.040**
Leisure activities	24 (26.4)	5 (45.5)	19 (23.8)	0.126
Personal relationships	13 (14.3)	4 (36.4)	9 (11.3)	**0.026**
Teasing	-	-	16 (20.0)	-
Sexual relationships	-	0 (0.0)	-	-
mDLQI scores				
Median	5	6	5	p = 0.668
IQR	3–8	3–9	3–8	

a only employed patients analyzed.

b n = 87.

c n = 7.

**Figure 2:**
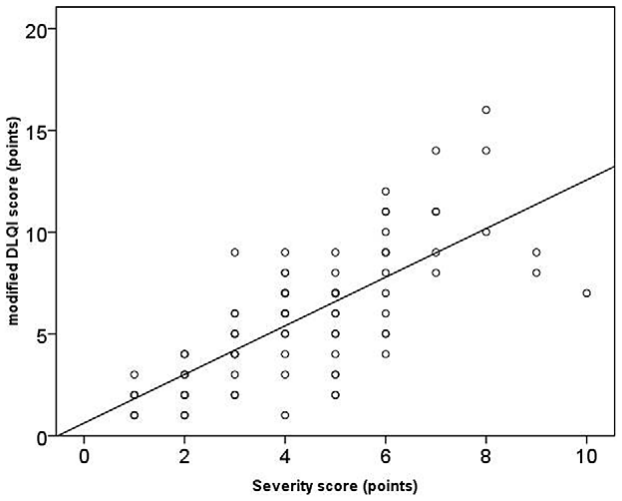
Correlation between severity of CLM and impairment of skin disease-associated life quality (rho = 0.76; p<0.001).

**Figure 3:**
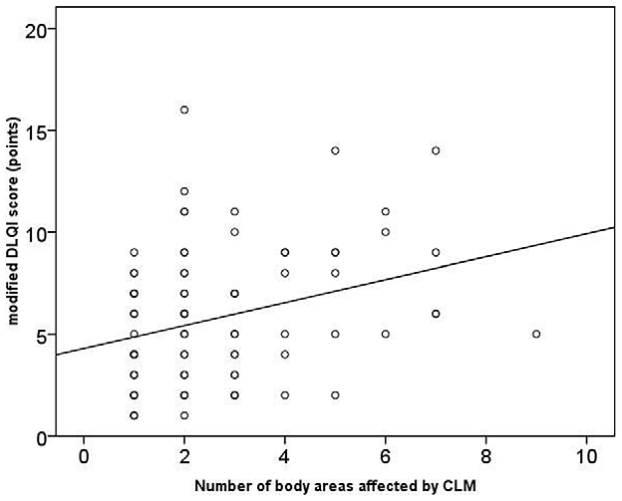
Correlation between the number of affected areas and impairment of skin disease-associated life quality (rho = 0.36; p = 0.004).

The degree of skin disease-associated life quality impairment correlated strongly with the severity of the infection (rho = 0.76; p<0.001) (Figure 2) and the number of affected body areas (rho = 0.30; p = 0.004) (Figure 3). A significant correlation existed between the presence of lesions in clearly visible body areas and the mDQLI score (p = 0.002).Skin disease-associated life quality impairment did not depend on the number of CLM episodes experienced previously (p = 0.88), the duration of the infection (p = 0.52), or the presence or absence of bacterial superinfection (p = 0.80).

**Figure 4:**
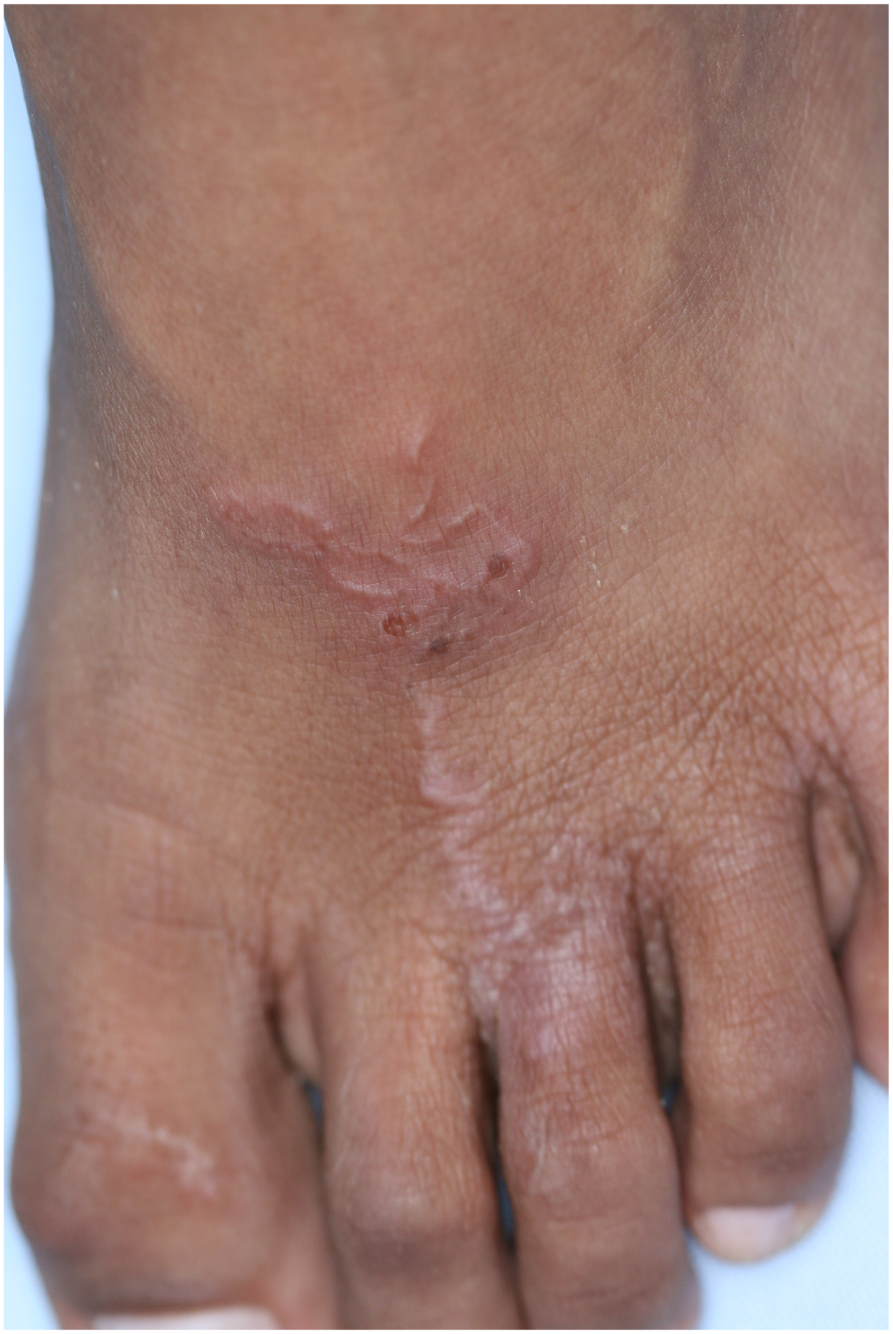
Excoriated lesion on the left foot of a 9-year-old boy, before treatment.

**Figure 5:**
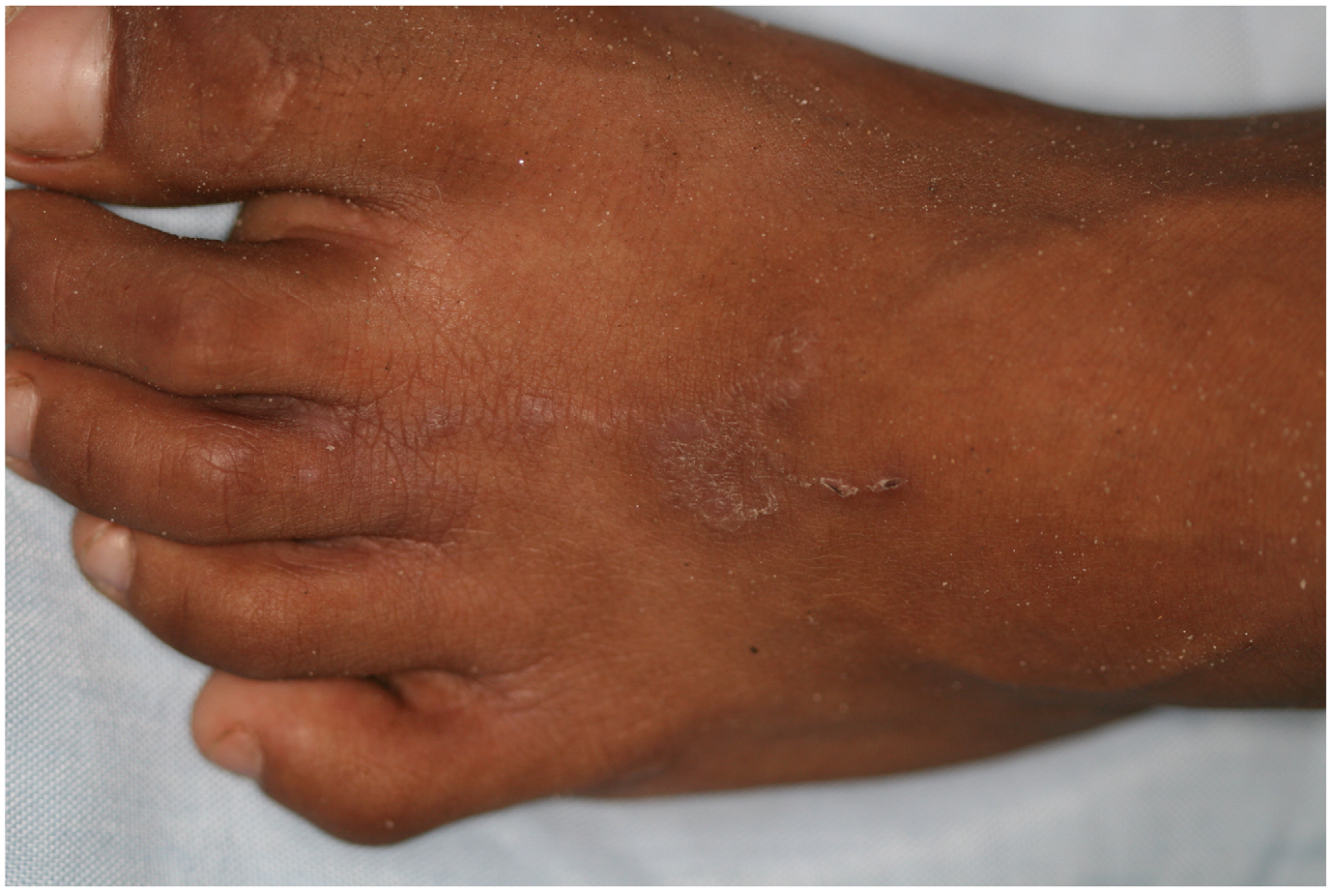
Excoriated lesion on the left foot of a 9-year-old boy, four weeks after treatment.

The follow–up examinations showed an improvement of skin disease-associated life quality two weeks after treatment with ivermectin (median mDLQI = 5 [IQR 3-8] versus 1 [IQR 0-3; p<0.001] Table 5). Four weeks after treatment, the median mDLQI score was zero and 82% of the patients reported a normalization of their skin disease-associated life quality. The normalization of skin disease-associated life quality was paralleled by a drastic reduction of the CLM severity score from a median of 4 points (IQR 3-6) to 1 point (IQR 1-1) two weeks after treatment with ivermectin and to 1 point (IQR 0-1) at the end of the study (both p<0.001). Figures 4 and 5 show the resolution of the inflammatory skin reactions around embedded hookworm larvae before and four weeks after treatment with ivermectin.

**Table 5:** Improvement of life quality after treatment with ivermectin.

Area of impairment	Significant impairment reported^a^			p-value before treatment versus	
	before treatment (n = 91)	2 weeks	4 weeks	2 weeks	4 weeks
		after treatment		after treatment	
		(n = 60)	(n = 55)		
Pruritus	85 (93.4)	21 ( 35.0)	12 (21.8)	**<0.001**	**<0.001**
Sleep disturbance	67 (73.6)	12 (20.0)	10 (18.3)	**<0.001**	**<0.001**
Feeling of shame	59 (64.8)	19 (31.7)	8 (14.5)	**<0.001**	**<0.001**
Need to dress differently	31 (34.0)	11 (18.3)	4 (7.3)	**0.018**	**0.018**
Problems faced at work/in school^b^	13 (16.5)	3 (3.4)	2 (2.3)	0.059	0.059
Leisure activities	24 (26.4)	11 (18.0)	11 (20.0)	0.168	0.637
Personal relationships	13 (14.3)	5 (8.3)	3 (5.5)	0.083	**0.014**
mDLQI scores					
Median	5	1	0	**<0.001**	**<0.001**
IQR	3–8	0–3	0–2		

a ≥2 points of the mDLQI.

b only employed patients analyzed.

## Discussion

Diseases of the skin lead to various levels of suffering. First, they cause defined clinical pathology, such as visible inflammation, pruritus or pain. Second, skin diseases are frequently chronic in nature and patients have to take drugs, either topically or orally, for a protracted period of time. Third, if gross alterations of the skin are located on visible body parts, they may, at worst, lead to social withdrawal and/or to exclusion from society, as it is the case, for instance, with leprosy [21]. Additionally, patients may be confronted with ignorance or misconceptions regarding the aetiology of their skin disease, such as the fear that the condition is contagious or related to poor personal hygiene – assumptions which may lead to stigmatisation [22], [23]. Lymphatic filariasis with gross lymphoedema is a paradigmatic example of this category of skin diseases [24], [25], [26].

CLM is an extremely itchy skin condition characterized by signs of inflammationm such as erythema. Since lesions are frequently located at visible body parts they are difficult to hide from the public [13] and negative impact on emotional well-being of the patient is possible..In our study 94.5% of patients with CLM reported reduction of their skin disease-associated life quality with a median mDLQI score of 5 (Table 3). The degree of skin disease-associated life quality impairment was positively correlated with the intensity of the infection (Figure 2), the number of body areas affected (Figure 3), and the presence of lesions at clearly visible body parts.

In contrast to a study in patients with scabies [20] we did not find different degrees of impairment between women and men. This could be due to the fact that scabies lesions usually are less obvious to the patient and external observers/third parties than highly inflamed larval tracks. Besides, in scabies the lesions are frequently located at “hidden” topographic areas, such as the interdigital spaces. Finally, the preponderance of male participants in the study – a consequence of the higher prevalence of CLM in males in the area where the study was conducted – may have blurred the differences between the sexes.

The most common finding associated with an impairment of skin disease-associated life quality was pruritus (93.4% of the patients). Pruritus causes the patient to scratch repeatedly- a behavior which does not pass unnoted by other members of society [27]. In addition, since the intensity of itching increases at night, it causes alterations in the sleep pattern. The affective aspect of pruritus may induce a vicious cycle in which increasing mental harm and distress lead to increased itching which, in turn, augments scratching [27], [28].

Insomnia was reported as a cause of life quality impairment by 73.6% of the patients. A previous study has shown that CLM related insomnia manifests itself as a sleep maintenance disorder [13], probably due to an increased perception of pruritus during the night. In patients with pruritus-induced perturbation of sleep, quality and duration of sleep are reduced as a consequence of shorter non-REM sleeping periods [29]. This may cause daytime somnolence, irritability and psychological problems such as anxiety disorders [30], [31].

It seems paradoxical that insomnia has been cited as most important restriction by people living in an *invasão*. From an outside observer's point of view, getting rest and sleep in this setting seems to be very difficult anyway: poor housing and a high population density allow noise to enter the house almost unaltered and loud music is heard even late at night. However, our patients seem to have adapted to the extremely noisy environment of an *invasão* and considered sleep and recreation to be severely impaired by the CLM-related pruritus. In fact, after treatment, insomnia was reduced significantly already after two weeks (Table 3).

The feeling of shame was noted by 64.8% of the patients. In our study on knowledge, attitudes and disease perception, it was found that shame frequently resulted from the concept that the occurrence of CLM reflects poor personal hygiene and lack of care (H. Lesshafft, unpublished observation).

Another commonly noted restriction is related to the necessity of patients with CLM to dress differently. In the hot climate of northern Brazil a great part of the body remains uncovered. Hence, skin lesions are difficult to hide and the effort to cover them up with extra clothes or bandages may lead to a reduction of self-esteem and provoke shame and stigmatisation [23], [32], [33]. These somato-psychological interactions were confirmed by our finding that mDLQI scores were highest in patients in whom lesions were present at clearly visible parts of the body.

Problems faced at work or at school and impairment of personal relationships were also a frequently noted restriction of skin disease-associated life quality (Table 4). Several mechanisms may underlie these psycho-social consequences. First, and similarly to other skin diseases such as psoriasis, the erroneous assumption that CLM is contagious leads to alterations in personal relationships and eventually to social exclusion [23]. Second, as shown in a previous focus group discussion in the study area (unpublished data), mothers frequently ban affected children from playing outside, partly to prevent a new infection and partly to avoid teasing by other children, which may cause boredom and or lead to a feeling of social exclusion. Thirdly, bullying and interrupted personal relationships may provoke a feeling of disgust and shame about the skin condition and reinforce an active withdrawal from social networks due to the fear of stigmatisation [23], [22].

With regard to personal relationships, the significantly lower impairment of skin disease-associated life quality in children compared to adults might be explained by the fact that consciousness about their own appearance interferes less in children's relationships. The higher impairment perceived by adults at work is presumably related to a similar mechanism. At work, adults are confronted with the “outside world” in which CLM reflects a life in poverty. In contrast, children - going to school in the community - do not leave their social environment and consequently may perceive less life quality impairment.

Hitherto, only a few studies have attempted to determine skin disease-associated life quality impairment in tropical parasitic skin diseases.

While in patients with active cutaneous leishmaniasis or onchocerciasis, the average impairment was found to be higher than in the CLM patients of our study, skin disease-associated life quality restrictions in lymphatic filariasis caused a similar or higher impairment depending on the severity of lymphoedema [24], [25], [26], [32], [33], [34]. In contrast, patients with scabies living in an invasão in Northeast Brazil percieved less impairment than our patients with CLM [20]. In scabies the duration of infection, but not the number of infested body areas, correlated with skin disease-associated life quality impairment. This is probably due to the rather slow development of the clinical pathology in scabies, where the degree of skin alteration increases gradually, whereas in CLM inflammatory skin reactions develop within a couple of days.

We think that our data clearly indicate a cause-effect relationship between cutaneous larva migrans and impaired quality of life. First, the severity of disease was significantly correlated to the degree of impaired quality of life (rho = 0.76; p<0.001) and number of body areas affected (rho = 0.30; p = 0.004), indicating positive “dose-response” relationships. Second, already two weeks after the regression of skin lesions due to treatment with ivermectin, the degree of life quality impairment decreased significantly. Taken together, these findings provide substantial evidence that the impairment of life quality is the consequence of the parasitic skin disease as it has been observed in patients suffering from other parasitic infections [24]–[26] [32]–[34]. These findings also suggest that a treatment that costs approximately 40–80 eurocents, not only abrogates clinical pathology, but also averts stressful psycho-social consequences and prevents the development of secondary morbidity when given promptly.

When interpreting our results one has to take into account that skin disease-associated life quality of people living in misery in an urban slum is very low per se [35]. Housing is poor, sanitary infrastructure is deficient, crowding is common and social problems such as unemployment, alcoholism, illiteracy, and violence prevail. Obviously, these characteristics should mitigate perceived restrictions on skin disease-associated life quality in our patients. In fact, the results of another study in the same setting showed that members of the community considered parasitic skin diseases negligible in comparison to the existential problems of daily life (H. Lesshafft, unpublished observation).

In conclusion, CLM impairs the physical and mental wellbeing as well as social interaction of patients in a setting where skin disease-associated life quality is generally low. A single dose of ivermectin caused a complete resolution of the lesions within one month and restored skin disease-associated life quality to the normal level.
